# SERS-Based Immunoassay for α-Fetoprotein Biomarker Detection Using an Au-Ag Nanostars Platform

**DOI:** 10.3390/bios15090632

**Published:** 2025-09-22

**Authors:** Josué Ismael García-Ramírez, Marcos Luna-Cervantes, Irma Yadira Izaguirre-Hernández, Julián Hernández-Torres, Enrique Juárez-Aguilar, Pablo Thomas-Dupont, José María Remes-Troche, Luis Zamora-Peredo

**Affiliations:** 1 Centro de Investigación en Micro y Nanotecnología, Universidad Veracruzana, Boca del Río 94294, Mexico; zs21023454@estudiantes.uv.mx (J.I.G.-R.);; 2 Facultad de Bioanálisis, Universidad Veracruzana, Veracruz 91700, Mexico; 3 Instituto de Ciencias de la Salud, Universidad Veracruzana, Xalapa-Enriquez 91190, Mexico; 4 Instituto de Investigaciones Médico Biológicas, Universidad Veracruzana, Veracruz 91700, Mexico

**Keywords:** liquid-phase SERS, cancer biosensor, alpha-fetoprotein, Au-Ag nanostars

## Abstract

Spiky Au-Ag nanostars offer intense plasmonic enhancement due to their sharp-tipped morphology, enabling powerful surface-enhanced Raman scattering (SERS). Here, we report a liquid-phase SERS platform that addresses current limitations in cancer biomarker detection, such as low sensitivity and dependence on Raman reporters. Nanostar concentration was tuned by simple centrifugation (10, 30, and 60 min), and their SERS performance was evaluated using methylene blue (MB) and mercaptopropionic acid (MPA) as probe molecules. Signal intensity scaled with nanostar content, enabling sensitive detection. Optimized nanostars were functionalized with MPA, 1-Ethyl-3-(3-dimethylamino1-Ethyl-3-(3dimethylaminopropyl1) carbodiimide (EDC), and N-Hydroxy succinimide (NHS) for covalent attachment of monoclonal anti-α-fetoprotein antibodies (AFP-Ab), facilitating the detection of AFP antigens across 167–38 ng/mL (antibody) and 500–0 ng/mL (antigen) ranges. The limit of detection (LOD) for the antigens was determined to be 16.73 ng/mL. Unlike conventional SERS systems, this aqueous, surfactant-free platform exploits the intrinsic vibrational modes of AFP, enabling sensitive and rapid biomarker detection with strong potential for early cancer diagnostics.

## 1. Introduction

Nowadays, the surface-enhanced Raman spectroscopy (SERS) technique with nanostructured substrates is widely recognized as a highly sensitive analytical method [1] in various areas of science, including agriculture, environmental, and medicine [2,3,4]. In the medical field, this technique has emerged as a diagnostic tool, identifying distinct vibrational modes in complex samples and facilitating the selective detection of individual analytes [5]. For example, in cancer diagnosis, reliable biomarker identification remains a major challenge due to the limited sensitivity of current devices, which often results in false negatives. Among different biomarkers, protein-based indicators—such as antibodies and antigens—are widely used [6,7], which include prostate-specific antigen (PSA), carcinoembryonic antigen (CEA), cancer antigen 125 (CA125), and α-fetoprotein (AFP) [8]. As a particular case, elevated levels of AFP in adults indicate certain pathological conditions that include different types of malignant neoplasms, such as liver cancer [9,10,11], and are also associated with ovarian, gastric, and lung cancers [12].

In recent years, several nanostructured platforms have been studied to improve AFP detection by SERS systems. Xinyu He designed Ag/Au nanocomposite chips capable of detecting AFP in healthy and diseased individuals [13]. On the other hand, Aonan Zhu later introduced a detection platform based on gold honeycomb nanostructures [14]. At the same time, Hao Ma reported a system using gold nanoparticles with dual signal amplification based on Raman frequency and intensity shifts [15]. Furthermore, most previous strategies rely on solid substrates with the use of Raman reporter molecules or complex immobilization steps, which limit their applicability in real-time or liquid-phase diagnostics.

Usually, SERS biosensors consist of a sandwich immunoassay using two antibodies: a capture antibody and a detection (or enhancement) antibody, with the antigen ‘sandwiched’ between them [16]. Recently, SERS-based AFP immunosensors using a monoclonal antibody for the best specific capture of the antigen and a polyclonal antibody anchored to metal nanoparticles to improve the sensitivity, where a Raman reporter is also linked, have been reported [17,18,19]. The monoclonal antibodies guarantee high molecular specificity, which leads to the minimization of cross-reactivity with structurally similar proteins in complex biological matrices such as serum. This selective recognition facilitates the effective localization of AFP molecules onto SERS-active regions, particularly at electromagnetic hot spots formed between nanostructures, where the Raman signal intensity is significantly enhanced. On the other hand, the use of monoclonal antibodies offers consistent binding affinity and epitope targeting, which improves the reproducibility and reliability of the detection system. Their well-defined functional groups also allow for stable and oriented surface immobilization on noble metal substrates, maintaining bioactivity and optimizing the proximity of the analyte to the enhancing surface. In general, the integration of AFP monoclonal antibodies is essential to achieve robust, sensitive detection based on the vibrational modes of AFP without the need to use external Raman reporters (methylene blue, Rhodamine, etc.) [18,20].

Among metallic nanostructures, Au-Ag nanostars have demonstrated exceptional SERS enhancement due to their sharp tips, where the electromagnetic field is concentrated, amplifying the Raman signal by several orders of magnitude [18,19]. However, despite this remarkable performance, studies using nanostar-based systems for AFP detection are currently still limited. For instance, J. Zhao conducted a SERS immunoassay using 4-mercaptobenzoic acid (4-MBA) as a Raman reporter and silicon-coated Au/Ag nanostars on a nitrocellulose membrane [17], obtaining a limit of detection (LOD) of 0.72 pg/mL and a wide and clinically relevant linear detection range from 3 pg/mL to 3 mg/mL. Recently, Kang Yang et al. proposed a facile immunoassay that used gold nanostar-labeled rabbit anti-AFP as a capture antibody and gold nanoparticle-conjugated goat anti-rabbit IgG as an enhancement antibody for the construction of a detection strategy for AFP analysis, but their characterization was limited to UV light absorption [21].

Building upon these efforts, the present study introduces an aqueous, reporter-free SERS platform based on Au-Ag nanostars functionalized with mercaptopropionic acid (MPA), 1-Ethyl-3-(3-dimethylamino1-Ethyl-3-(3dimethylaminopropyl1) carbodiimide (EDC), and N-Hydroxy succinimide (NHS). SERS technology was used for the detection of the AFP biomarker through covalent immobilization of anti-AFP monoclonal antibodies (AFP-Ab) using EDC/NHS coupling, followed by specific recognition of the AFP antigen. This reporter-free system operates entirely in the liquid phase by monitoring the intrinsic vibrational modes of biomolecular interactions without relying on solid substrates or Raman reporter molecules. The proposed approach provides a sensitive and scalable strategy for AFP detection, with strong potential for early-stage cancer diagnostics.

## 2. Materials and Methods

### 2.1. Materials

Tetrachloroauric acid (HAuCl_4_·3H_2_O), silver nitrate (AgNO_3_), L-ascorbic acid, polyvinylpyrrolidone wt. 40,000 (PVP), mercaptopropionic acid (MPA), 1-Ethyl-3-(3-dimethylamino1-Ethyl-3-(3dimethylaminopropyl1) carbodiimide (EDC), N-Hydroxy succinimide (NHS), and bovine serum albumin (BSA) were purchased from Sigma-Aldrich (St. Louis, MO, USA), sodium chloride (NaCl; Meyer, Mexico), monoclonal anti-α-fetoprotein antibodies (AFP-Ab; MyBioSource, San Diego, CA, USA), and α-fetoprotein antigens (MexLab, Jalisco, Mexico).

### 2.2. Synthesis of Nanostars

For the specific fabrication of Au-Ag nanostars, solutions of 0.25 mM tetrachloroauric acid (HAuCl_4_·3H_2_O), 1 M sodium chloride (NaCl), and 3 mM silver nitrate (AgNO_3_) were prepared. Following this step, ascorbic acid at a concentration of 100 mM was added and mixed, immediately followed by adding 0.1% (*w*/*w*) polyvinylpyrrolidone (PVP). Finally, the samples were stored at 4 °C. The samples were transferred to vials and cleaned with deionized water, performed at 7000 rpm for 10 min, where the excess solution was removed, leaving only the nanostar concentrate. This process was repeated one, three, and six times, yielding 10, 30, and 60 min sample groups.

### 2.3. SERS Evaluation of Au-Ag Nanostars Using MB and MPA

To evaluate the SERS performance of the nanostar samples, two probe molecules, methylene blue (MB) and mercaptopropionic acid (MPA), were used. For MB analysis, 20 µL of each nanostar concentrate was mixed with 10 µL of MB at concentrations of 1 × 10^−5^ M (High-MB), 1 × 10^−6^ M (Mid-MB), and 1 × 10^−7^ M (Low-MB). All measurements were performed in the liquid phase without incubation. For MPA-based analysis, 20 µL of each nanostar sample was combined with 10 µL of MPA at concentrations of 1 × 10^−3^ M (High-MPA), 1 × 10^−4^ M (Mid-MPA), and 1 × 10^−5^ M (Low-MPA), followed by a 1 h incubation prior to SERS measurement.

### 2.4. Bioconjugation of Au-Ag Nanostars with AFP Antibodies and Antigens

The nanostars were functionalized using MPA at Mid-MPA concentration (1 × 10^−4^ M) with an incubation time of 1 h. Subsequently, EDC and NHS were added at 10 mM each and incubated for 30 min to activate the carboxyl groups. The AFP-Ab were added at concentrations of 167, 100, 71, 56, 45, and 38 ng/mL, followed by a 30 min incubation and washing at 7500 rpm for 10 min. Subsequently, the samples were treated with bovine serum albumin (BSA) for blocking and washed. The samples were then analyzed using SERS and UV-Vis spectroscopy. Afterwards, the AFP antigen was added at 500, 250, 50, and 0 ng/mL, followed by a 30 min incubation. Finally, the samples were washed and subjected to SERS and UV-Vis analysis.

### 2.5. Optical and Morphological Characterization

SERS measurements were carried out using an Ocean Insights QE Pro Raman spectrometer with a 785 nm laser. Optical properties were assessed by Thermo Scientific Genesys 50 UV-Vis spectrophotometer in the 350–900 nm range. Nanostar concentrates were tested for SERS evaluation by adding MB at High-MB, Mid-MB, and Low-MB levels without incubation. Nanostar morphology and EDS were characterized using a JEOL JSM-7600F Field Emission Scanning Electron Microscope.

## 3. Results and Discussion

**Figure 1:**
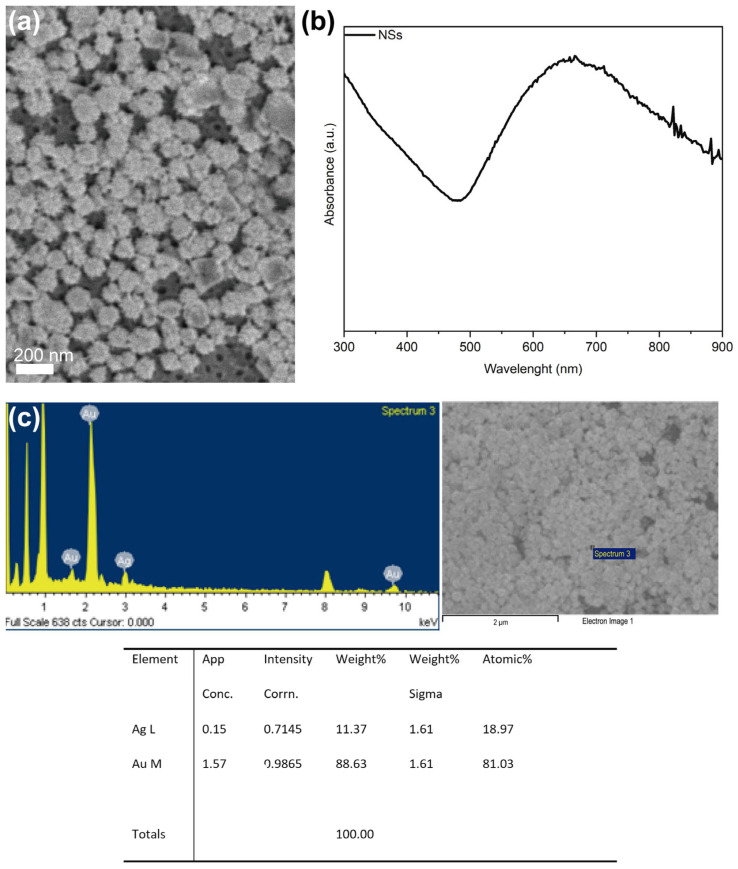
(**a**) SEM image of Au-Ag nanostars showing sharp-tipped morphology. (**b**) UV-Vis absorption spectra of nanostar samples, confirming their plasmonic behavior in the 300–900 nm range, and (**c**) EDS-SEM analysis.

**Figure 2:**
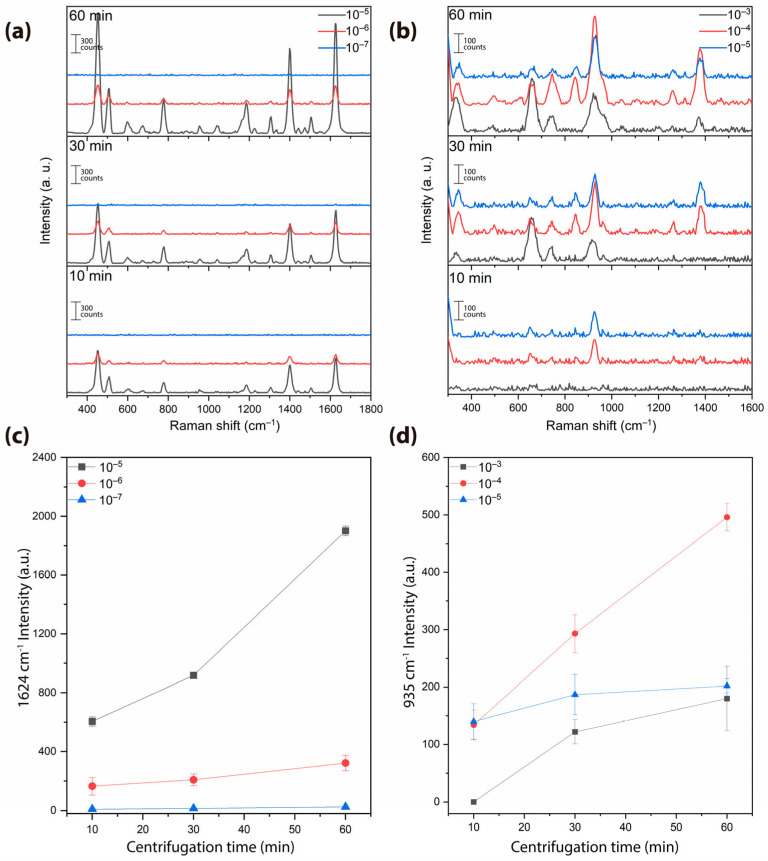
(**a**) SERS spectra of methylene blue (MB) at High-MB (1 × 10^−5^ M), Mid-MB (1 × 10^−6^ M), and Low-MB (1 × 10^−7^ M) concentrations for Au-Ag nanostar samples from the 10 min, 30 min, and 60 min groups. (**b**) SERS spectra of mercaptopropionic acid (MPA) at High-MPA (1 × 10^−3^ M) (black square), Mid-MPA (1 × 10^−4^ M) (red dot), and Low-MPA (1 × 10^−5^ M) (blue triangle) concentrations for the same sample groups. (**c**,**d**) Intensity of the 1624 cm^−1^ MB (**c**) and 935 cm^−1^ MPA (**d**) vibrational modes as a function of centrifugation time.

The SEM image in Figure 1a shows nanostars with diameters between 150 and 180 nm, with tip lengths between 20 and 30 nm. UV-Vis absorption in Figure 2b confirmed the presence of nanostars in the solution, showing broad plasmonic bands consistent with the Au-Ag structures described previously [22,23,24,25,26,27,28,29,30,31,32,33]. As described in the literature [24,34,35], polyvinylpyrrolidone (PVP) acts as a stabilizer by adsorbing onto the nanostar surface, preventing aggregation and preserving colloidal stability. We performed EDS-SEM analysis, which showed that the nanostars are an alloy of gold and silver [36]. The estimated atomic ratio was ~80% Au and 20% Ag.

As an initial strategy for analyte identification via Raman spectroscopy, MB was employed in conjunction with star-shaped nanostructures, owing to its well-defined vibrational modes that enhance spectral reproducibility. Its high chemical stability and strong affinity for metallic surfaces enable efficient adsorption onto gold and silver nanostructures, thereby facilitating optimal coupling with surface plasmons. This interaction significantly enhances the Raman signal intensity, making MB a suitable molecular probe for evaluating the performance of the SERS substrate [22,23]. SERS measurements using MB identified characteristic vibrational modes at 450, 504, 675, 772, 889, 954, 1039, 1074, 1185, 1303, 1399, 1505, and 1624 cm^−1^, in agreement with values of previous studies [37,38,39,40,41]. As shown in Figure 2a, the 10 min, 30 min, and 60 min samples were tested with High-MB and Mid-MB concentrations. Signal intensity increased with nanostar content, indicating a direct correlation between nanostructure density and electromagnetic field enhancement. At Low-MB concentration (1 × 10^−7^ M), no signal was detected in any sample. The 1624 cm^−1^ peak intensity, plotted in Figure 2c, highlights the superior SERS activity of the 60 min group. The analytical enhancement factor (AEF) was calculated for methylene blue with a concentration of 1 × 10^−4^ M with an intensity of 93.5 a.u. and an intensity of 23 a.u. for a concentration of 1 × 10^−8^ M. It should be noted that the measurement for these samples was carried out immediately, which influences the adsorption of the molecule and therefore its AEF.

The MPA was selected due to its bifunctional nature, featuring a thiol group (–SH) that enables covalent bonding with metallic surfaces such as gold and silver, and a carboxyl group (–COOH), which can be utilized for the immobilization of biomolecules, including antibodies and antigens [24,25]. MPA interaction with the nanostar surface was also evaluated. Vibrational modes at 661, 739, 935, 1294, and 1415 cm^−1^ were detected, consistent with the interaction and bonding between MPA and Ag [42,43,44,45,46,47]. Notably, no signal was observed for the 10 min group at High-MPA, likely due to nanoparticle destabilization by MPA acidity at high concentrations. In contrast, clear SERS signals were obtained for Mid-MPA and Low-MPA, particularly in the 30 min and 60 min groups, where increased nanostar density improved field localization (Figure 2b,d). The 935 cm^−1^ mode intensity followed the same trend, confirming effective analyte–nanostar interaction.

Following MPA optimization, the samples were functionalized with MPA, EDC, and NHS. It is known that the capture of antigens through the use of antibodies (immunoassays) immobilized on surfaces of metallic nanostructures has been widely used for the generation of biosensors that seek to improve the early diagnosis of cancer [26,27].

**Figure 3:**
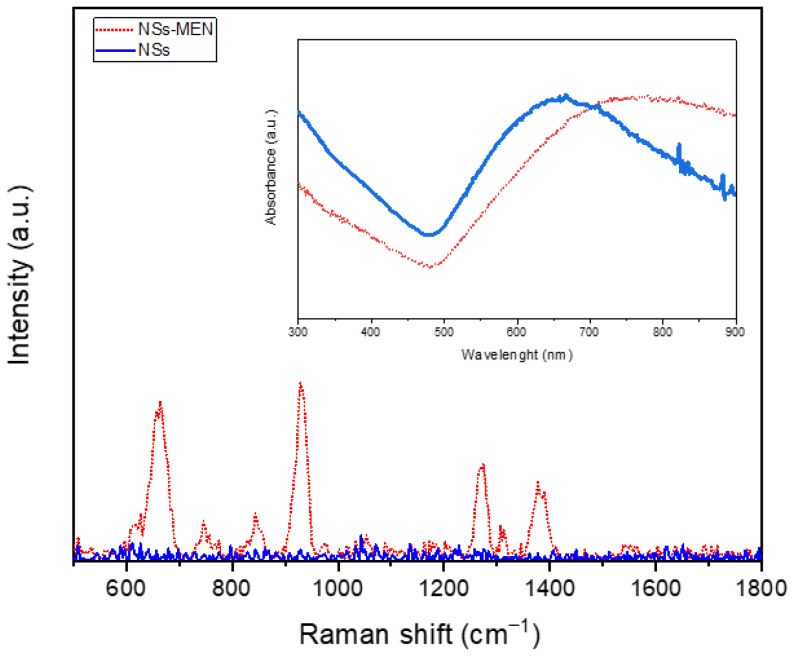
SERS spectra obtained from Au-Ag nanostars functionalized with MPA, EDC, and NHS (MEN), accompanied by the absorption spectra of the nanostructures before and after the functionalization process.

This process was carried out in two main stages. In the first stage, self-assembled monolayers (SAMs) were formed by anchoring a monolayer onto the surface of the nanostars. MPA was used as a functionalizing agent due to its ability to form stable bonds on metal surfaces. In the second stage, the surface was chemically activated with EDC and NHS for the coupling of biomolecules [26]. The activation of the carboxyl groups in the SAM by EDC and NHS generates a surface capable of forming covalent bonds with the amino groups of the biomolecule [27]. Raman measurements were performed on the functionalized samples, identifying the characteristic vibrational modes of the compounds. Figure 3 shows the vibrational modes located at 663, 745, 843, 928, 1270, and 1377 cm^−1^ of the nanostars with the SAM. These vibrational modes are attributed to the MPA, EDC, and NHS on the surface of the nanostars (NSs-MEN). The presence of these bands in the spectrum confirms the formation of covalent bonds between the carboxyl groups of MPA, EDC, and NHS [1,2,3,4,5,6]. Finally, the inset of Figure 3 shows the absorption spectra of the nanostars before and after functionalization with MPA, EDC, and NHS, where a red shift in the absorption spectrum is observed, attributed to the adsorption of these molecules on the surface of the nanostructures. Furthermore, this type of spectral shift is known to be consistent with the formation of molecular layers at the metal interface [48,49].

**Figure 4:**
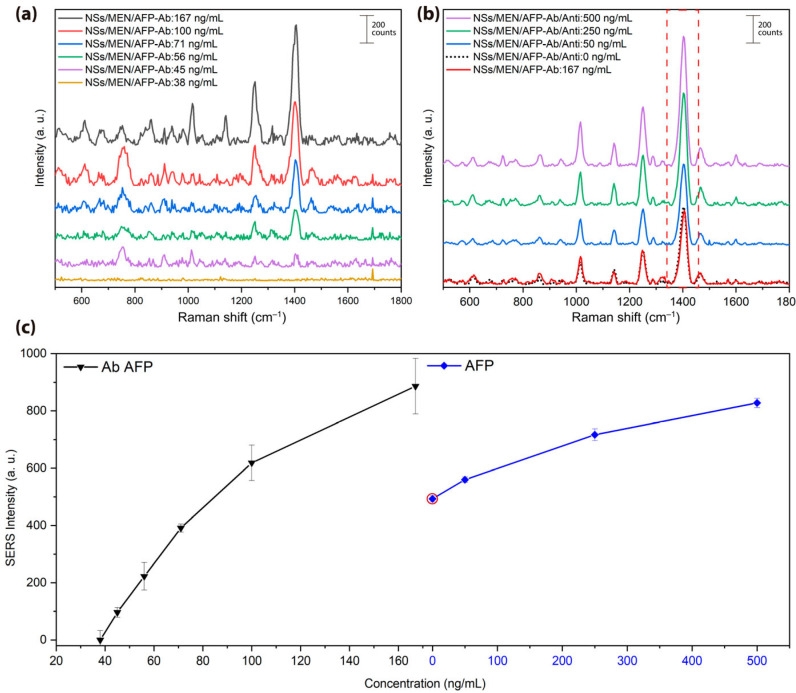
(**a**) SERS spectra of Au-Ag nanostars functionalized with antibodies at concentrations of 167, 100, 71, 56, 45, and 38 ng/mL. (**b**) SERS spectra of the same nanostars after incubation with AFP antigens at 500, 250, 50, and 0 ng/mL, the red box highlights the vibrational mode at 1405 cm^−1^, which is characteristic of both antibodies and antigens. (**c**) Intensity of the 1405 cm^−1^ vibrational mode as a function of antibody and antigen concentration, and the red dot represents the persistent Raman signal attributable to the antibody.

After functionalization with MPA, EDC, and NHS, alpha-fetoprotein antibodies, AFP-Ab (NSs/MEN/AFP-Ab), were added at various concentrations (167, 100, 71, 56, 45, and 38 ng/mL). The vibrational modes of the antibody are shown in Figure 4a as well as in Table 1, where the key vibrational modes and their biomolecular assignments are summarized [28,37,38]. Meanwhile, the vibrational modes at 611, 1015, and 1600 cm^−1^ can be attributed to phenylalanine, an amino acid present in proteins [39]. The bands at 290 and 1600 cm^−1^ suggest the contribution of tyrosine and tryptophan, indicating protein composition [40,50]. In addition, the bands observed at 1251, 1570 cm^−1^ correspond to secondary structural elements present in proteins, which may be associated with the antibodies and antigens added in the assay [42,43]. Likewise, the peaks at 728, 1290, 1322, and 1486 cm^−1^ can be associated with lipid components from cell membranes or residual contaminants [44]. Other authors associate the modes at 1322 and 1570 cm^−1^, which are linked to nitrogenous bases such as adenine and guanine, suggesting the presence of DNA [51,52]. Finally, the band at 1405 cm^−1^ can be assigned to the carboxylate group (COO^−^) [51,52,53,54,55,56], associated with proteins and thus with the antibodies and antigens included in the assay. This vibrational mode, related to COO^−^ deformation and amide functional groups, was an indicator of protein adsorption. The SERS signal decreased with lower antibody concentration, with 45 ng/mL as the lowest detectable limit in this system. The sample functionalized with 167 ng/mL of AFP-Ab was used as the reference for antigen detection due to its highest baseline SERS signal. Subsequent incubation with AFP antigens (NSs/MEN/AFP-Ab/Anti) at 500, 250, 50, and 0 ng/mL concentrations resulted in a change in SERS intensity (Figure 4b). These variations are attributed to antigen–antibody binding on the nanostar surface. The SERS substrate was functionalized with a fixed amount of anti-AFP antibodies, followed by the addition of antigen standards at increasing concentrations, including the zero standard (absence of antigen). After the incubation and washing steps corresponding to the zero standard, a persistent Raman signal attributable to the antibody was observed, which is highlighted within the red box in Figure 4b and indicated by the red dot in Figure 4c. This baseline signal originates from the vibrational fingerprint of the antibody itself, which contributes characteristic bands upon its immobilization on the gold nanostructured surface. The covalent attachment is achieved through the formation of SAMs using MPA and chemical activation with EDC/NHS, ensuring the antibody’s stability at the metal interface and its effective coupling with the SERS hotspots. The subsequent increase observed after antigen addition is therefore attributed to antigen–antibody complex formation, which further stabilizes and reorganizes the biomolecular layer at the interface, thereby enhancing the spectral signal obtained. This configuration allows the characteristic vibrational modes of the antibody, particularly those associated with aromatic residues such as phenylalanine, tyrosine, and tryptophan, to remain detectable in the Raman spectrum even in the absence of the antigen [57,58,59,60,61,62,63]. Furthermore, since both the antibody and the AFP antigen are proteins, they share a similar chemical composition, which results in overlapping vibrational modes in their respective spectrum. This spectral similarity may complicate direct differentiation between the two; however, it does not preclude it, as specific antigen–antibody interactions can induce conformational changes or activate additional vibrational modes that enable their distinction with sufficient spectral resolution [64,65,66].

Figure 4c shows the intensity trend of the 1405 cm^−1^ mode across antigen concentrations. Interestingly, this signal increased with higher AFP levels, suggesting that the formation of the antigen–antibody complex may reorganize or stabilize surface binding sites, thereby enhancing the SERS response. This response in signal intensity may be due to the increase in antigens, since, as they are the same protein blocks, they contribute to the same vibrational modes as the antibody, thus increasing the signal in the spectra. As shown in Figure 4c, the 1405 cm^−1^ vibrational mode is consistently present in the samples, corresponding to the symmetric stretching of the carboxylate group (COO^−^) in proteins. This feature reflects the covalent immobilization of antibodies at the gold surface and becomes more intense upon antigen addition, suggesting reinforcement of the biomolecular layer after antigen–antibody binding. Other vibrational modes are observed for the system with antibodies and antigens at 1251 and 1570 cm^−1^, associated with amide III and amide II. For the mode at 1605 cm^−1^ attributed to the amide I band, this vibrational mode was not only detectable for the samples exclusively with the AFP antibody, but when adding the antigen to the system, the Raman intensity increases, which suggests its presence.

The limit of detection (LOD) of the antigen was determined using the Raman intensity of the 1405 cm^−1^ vibrational mode in the analyzed samples. The calculation was based on the following equation [67]:

(1) $$LOD=\frac{{(I}_{ref}-B)}{M}$$

where M represents the slope and *B* the intercept obtained from the linear regression of the calibration curve (SERS intensity vs. antigen concentration). The reference intensity (*I_ref_)* was defined as the signal of the blank (zero standard) adjusted by the variability of the measurements, according to the following:

(2) $$I_{ref}=I_{blank}+3\sigma$$

Here, *I_blank_* corresponds to the SERS intensity of the blank sample, and *σ* is the standard deviation of blank measurements. Thus, the criterion of *I_blank_ +* 3*σ* ensures that the LOD corresponds to the lowest antigen concentration that produces a measurable signal significantly above the statistical variation in the blank. Based on the calculations, a limit of detection of 16.73 ng/mL was determined for antigen detection.

Although our system does not present such a low detection limit as some works presented by [68,69,70], it is important to note that most of them depend on solid substrates, complex systems, or the use of Raman reporter molecules, which limits their scalability and applicability in the liquid phase. For its part, our approach introduces an aqueous SERS platform, free of Raman reporters, based on Au–Ag nanostars functionalized with MPA, EDC, and NHS. The covalent immobilization of anti-AFP antibodies and the specific recognition of the antigen is generated in a liquid phase, directly monitoring the vibrational modes of biomolecular interactions, without the need for labels, which makes it a promising system for the detection of AFP.

**Figure 5:**
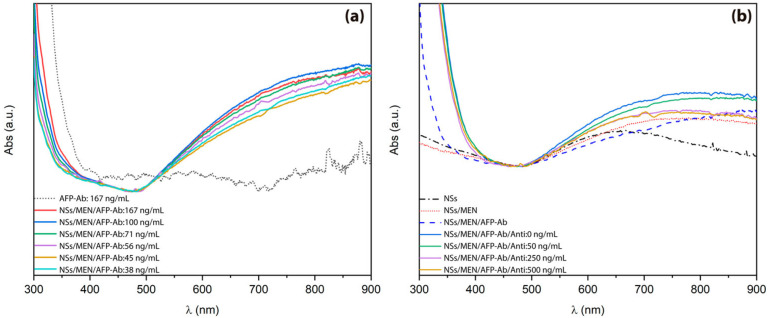
(**a**) UV-Vis absorption spectrum of Au-Ag nanostars functionalized with MPA, EDC, NHS, and anti-AFP antibodies (NSs/MEN/AFP-Ab). (**b**) Absorption spectrum of the same nanostars after incubation with AFP antigens (NSs/MEN/AFP-Ab/Anti) at different concentrations.

The samples prepared and analyzed by SERS were also characterized by ultraviolet-visible (UV-Vis) spectroscopy. This analysis was used to monitor variations in the surface plasmon band of the nanostars during the MPA functionalization processes, as well as activation with EDC-NHS and subsequent biofunctionalization with antibodies and AFP antigens. At each stage, it was possible to observe shifts in the absorption band toward longer wavelengths, demonstrating the progressive incorporation of each molecular building block into the surface of the nanostructures, thus acting as a complementary test to the results obtained by SERS. For this analysis, a standard concentration of 167 ng/mL of anti-alpha-fetoprotein antibodies was used as a reference (Figure 5a). The morphology and dimensions of Au-Ag nanostars play a fundamental role in determining the position and intensity of their optical absorption bands, mainly due to their influence on localized surface plasmon resonances. Furthermore, dipolar electrostatic interactions between adjacent nanostructures and target molecules, along with specific molecules (such as antibody–antigen recognition binding on the nanostar surface), can induce measurable changes in the absorption spectrum. These effects are strongly dependent on the interparticle distance and surface conditions [51,58]. AFP antigen concentrations of 0, 50, 250, and 500 ng/mL were evaluated, revealing that increasing the concentration of alpha-fetoprotein (AFP) leads to a progressive decrease in the system’s absorbance (Figure 5b). This behavior is attributed to nanostar aggregation induced by immunocomplex formation [67,68,69,70,71,72].

## 4. Conclusions

In this study, we developed a SERS platform based on Au–Ag nanostars for the detection of the cancer biomarker AFP. By adjusting the centrifugation time during synthesis, we tuned the nanostar concentration and obtained optimal SERS enhancement using MB and MPA as probe molecules. The system allowed the detection of AFP antibodies and antigens in the liquid phase. Characteristic vibrational modes were identified across different concentrations, and UV–Vis spectra confirmed molecular interactions on the nanostar surface. The limit of detection (LOD) of the antigens was determined to be 16.73 ng/mL. These results demonstrate the proof-of-concept applicability of Au–Ag nanostars as biosensing elements for AFP detection, while further studies with real and spiked samples will be required to evaluate their potential in clinical diagnostics.

## Figures and Tables

**Table 1:** Vibrational modes observed in the synthesized samples and their assignment based on reports in the literature.

Peak Position (cm^−1^)	Assignment
611	ω ν(C–C), τ phenylalanine, protein
728	ω ν(C–S, C–C), protein, CH_2_ rocking, C–N (membrane phospholipid head)
884	ω backbone, proteins C–C skeletal
1015	S ν(C–C), symmetric ring breathing mode of phenylalanine
1141	M ν(C–N, C–C), skeletal
1251	S amide III
1290	S CH_3_CH_2_, τ of lipids, collagen, tryptophan
1322	S CH_3_CH_2_, γ of collagen and polynucleotide chain (DNA bases)
1405	COO-
1468	VS δ(CH_2_), lipids, ν(C–H), proteins (collagen)
1570	S DNA, adenine, guanine, C=C, δ(N–H), ν(C–N), amide II
1600	S ν(C=C), phenylalanine, tyrosine, amide I

Notation: S—strong, M—medium, VS—very strong, ω—weak, ν—stretching vibration, τ—twisting, γ—wagging, δ—deformation.

## Data Availability

Data is contained within the article.

## References

[1] Vo-Dinh T., Wang H., Scaffidi J. (2010). Plasmonic Nanoprobes for SERS Biosensing and Bioimaging. J. Biophotonics.

[2] Guo Z., Chen P., Yosri N., Chen Q., Elseedi H., Zou X., Yang H. (2023). Detection of Heavy Metals in Food and Agricultural Products by Surface-Enhanced Raman Spectroscopy. Food Rev. Int..

[3] He J., Li X., Li J. (2022). Facile Construction of Silver Nanocubes/Graphene Oxide Composites for Highly Sensitive SERS Detection of Multiple Organic Contaminants by a Portable Raman Spectrometer. J. Environ. Chem. Eng..

[4] Tripathi M., Singh K., Yadav U., Srivastava R., Gagwar M., Nath G., Saxena P., Srivastava A. (2022). SERS Based Rapid and Ultrasensitive Detection of Japanese Encephalitis Virus. Antivir. Res..

[5] Blanco-Formoso M., Alvarez-Puebla R. (2020). Cancer Diagnosis through SERS and Other Related Techniques. Int. J. Mol. Sci..

[6] Vázquez-Iglesias L., Casagrande G., García-Lojo D., Leal L., Ngo T., Pérez-Juste J., Reis R., Kant K., Pastoriza-Santo I. (2024). SERS Sensing for Cancer Biomarker: Approaches and Directions. Bioact. Mater..

[7] Wu L., Qu X. (2015). Cancer Biomarker Detection: Recent Achievements and Challenges. Chem. Soc. Rev..

[8] Devi R., Doble M., Verma R. (2015). Nanomaterials for Early Detection of Cancer Biomarker with Special Emphasis on Gold Nanoparticles in Immunoassays/Sensors. Biosens. Bioelectron..

[9] Zhang J., Chen G., Zhang P., Zhang J., Li X., Gan D., Cao X., Du H., Ye Y. (2020). The threshold of alpha-fetoprotein (AFP) for the diagnosis of hepatocellular carcinoma: A systematic review and meta-analysis. PLoS ONE.

[10] Yeo Y., Lee Y., Tseng H., Zhu Y., You S., Agopian V., Yang J. (2024). Alpha-fetoprotein: Past, present, and future. Hepatol. Commun..

[11] Lu Y., Lin B., Li M. (2024). The role of alpha-fetoprotein in the tumor microenvironment of hepatocellular carcinoma. Front. Oncol..

[12] Zhan Z., Chen B., Yu J., Zheng J., Zheng Y., Sun M., Peng L., Guo Z., Wang X. (2022). Elevated Serum Alpha-Fetoprotein Is a Significant Prognostic Factor for Patients with Gastric Cancer: Results Based on a Large-Scale Retrospective Study. Front. Oncol..

[13] He X., Ge C., Zheng X., Tang B., Chen L., Li S., Wang L., Zhang L., Xu Y. (2020). Rapid Identification of Alpha-Fetoprotein in Serum by a Microfluidic SERS Chip Integrated with Ag/Au Nanocomposites. Sens. Actuators B Chem..

[14] Zhu A., Zhao X., Cheng M., Chen L., Wang Y., Zhang X., Zhang Y., Zhang X. (2019). Nanohoneycomb Surface-Enhanced Raman Spectroscopy-Active Chip for the Determination of Biomarkers of Hepatocellular Carcinoma. Appl. Mater. Interfaces.

[15] Ma H., Sun X., Chen L., Cheng W., Han X., Zhao B., He C. (2017). Multiplex Immunochips for High-Accuracy Detection of AFP-L3% Based on Surface-Enhanced Raman Scattering: Implications for Early Liver Cancer Diagnosis. Anal. Chem..

[16] Pollap A., Swit P. (2022). Recent Advances in Sandwich SERS Immunosensors for Cancer Detection. Int. J. Mol. Sci..

[17] Zhao J., Wu C., Zhai L., Shi X., Li X., Weng G., Zhu J., Li J., Zhao J. (2019). A SERS-Based Immunoassay for the Detection of α-Fetoprotein Using AuNS@ Ag@SiO2 Core–Shell Nanostars. J. Mater. Chem. C.

[18] Er E., Sánchez-Iglesias A., Silvestri A., Arnaiz B., Liz-Marzán L., Prato M., Criado A. (2021). Metal Nanoparticles/MoS2 Surface-Enhanced Raman Scattering Based Sandwich Immunoassay for α Fetoprotein Detection. Appl. Mater. Interfaces.

[19] Li J., Fang C., Yao Y., Chen L., Lin B., Wang Y., Guo L. (2024). Rapid antibody conjugation strategy via instant charge inversion of AuNBPs toward ultrasensitive SERS-LFIA detection of AFP. Microchem. J..

[20] Uotila M., Ruoslahti E., Engvall E. (1981). Two-Site Sandwich Enzyme Immunoassay with Monoclonal Antibodies to Human Alpha-Fetoprotein. J. Immunol. Methods.

[21] Yang K., Yang F., Lu X., Li H., Yang Z., Yin Q., Zhang L., Long Y., Shen C., Chen L. (2025). Facile Immunoassay Constructed by Gold Nanostar-Labeled Rabbit-AFP Antibody and Gold Nanoparticle-Conjugated Goat Anti-Rabbit IgG. Nanomaterials.

[22] Koutsompeli E., Murray J., Langford D., Bon R., Johnson S. (2015). Probing molecular interactions with methylene blue derivatized self-assembled monolayers. Sens. Bio-Sens. Res..

[23] Bollinger J.-C., Lima E., Mouni L., Savestrini S., Nguyen H. (2025). Molecular properties of methylene blue, a common probe in sorption and degradation studies: A review. Environ. Chem. Lett..

[24] Chah S., Yi J., Pettit C., Roy D., Fendler J. (2002). Ionization and Reprotonation of Self-Assembled Mercaptopropionic Acid Monolayers Investigated by Surface Plasmon Resonance Measurements. Langmuir.

[25] Kudelski A. (2002). Raman study on the structure of 3-mercaptopropionic acid monolayers on silver. Surf. Sci..

[26] Smolsky J., Kaur S., Hayashi C., Batra S., Krasnslobodtsev A. (2017). Surface-Enhanced Raman Scattering-Based Immunoassay Technologies for Detection of Disease Biomarkers. Biosensors.

[27] Lin C., Li Y., Peng Y., Zhao S., Xu M., Zhang L., Huang Z., Shi J., Yang Y. (2023). Recent development of surface-enhanced Raman scattering for biosensing. J. Nanobiotechnol..

[28] Xu J., Yu T., Zois C., Cheng J.-X., Tang Y., Harris A., Huang W. (2021). Unveiling Cancer Metabolism through Spontaneous and Coherent Raman Spectroscopy and Stable Isotope Probing. Cancers.

[29] Ye Z., Li C., Celentano M., Lindley M., O’Reilly T., Greer A., Huang Y., Hardacre C., Haigh S., Xu Y. (2021). Surfactant-Free Synthesis of Spiky Hollow Ag–Au Nanostars with Chemically Exposed Surfaces for Enhanced Catalysis and Single-Particle SERS. JACS Au.

[30] Pei Y., Wang Z., Zong S., Cui Y. (2013). Highly Sensitive SERS-Based Immunoassay with Simultaneous Utilization of Self-Assembled Substrates of Gold Nanostars and Aggregates of Gold Nanostars. J. Mater. Chem. B.

[31] Barbosa S., Agrawal A., Rodríguez-Lorenzo L., Pastoriza-Santos I., Alvarez-Puebla R., Kornowski A., Weller H., Liz-Marzán L. (2010). Tuning Size and Sensing Properties in Colloidal Gold Nanostars. Langmuir.

[32] Cheng L.-C., Huang J.-H., Chen H., Lai T.-C., Yang K.-Y., Liu R.-S., Hsiao M., Chen C.-H., Her L.-J., Tsai D. (2012). Seedless, silver-induced synthesis of star-shaped gold/silver bimetallic nanoparticles as high efficiency photothermal therapy reagent. J. Mater. Chem..

[33] Šubr M., Procházka M. (2018). Polarization-and Angular-Resolved Optical Response of Molecules on Anisotropic Plasmonic Nanostructures. Nanomaterials.

[34] Wang Y., Serrano A., Sentosun K., Bals S., Liz-Marzán L. (2015). Stabilization and Encapsulation of Gold Nanostars Mediated by Dithiols. Small.

[35] Liebig F., Henning R., Sarhan R., Prietzel C., Schmitt C., Bargheer M., Koetz J. (2019). A Simple One-Step Procedure to Synthesise Gold Nanostars in Concentrated Aqueous Surfactant Solutions. RSC Adv..

[36] Pham T., Vu X., Dien N., Trang T., Van Truong N., Thanh T., Tan P., Ca N. (2020). The Structural Transition of Bimetallic Ag–Au from Core/Shell to Alloy and SERS Application. RSC Adv..

[37] Sharaha U., Hania D., Lapidot I., Salman A., Huleihel M. (2023). Early Detection of Pre-Cancerous and Cancerous Cells Using Raman Spectroscopy-Based Machine Learning. Cells.

[38] Makki A., Massot V., Byrne H., Respaud R., Bertrand D., Mohammed E., Chourpa I., Bonnier F. (2021). Understanding the discrimination and quantification of monoclonal antibodies preparations using Raman spectroscopy. J. Pharm. Biomed. Anal..

[39] Hernández B., Pfluger F., Adenier A., Kruglik S., Ghomi M. (2010). Vibrational Analysis of Amino Acids and Short Peptides in Hydrated Media. VIII. Amino Acids with Aromatic Side Chains: L-Phenylalanine, L-Tyrosine, and L-Tryptophan. J. Phys. Chem. B.

[40] Ramirez-Perez J., Durigo D. (2022). Surface-Enhanced Raman Spectroscopy (SERS) for characterization SARS-CoV-2. J. Saudi Chem. Soc..

[41] Yang J., Chen B., Peng J., Huang B., Deng W., Xie W., Luo Z. (2021). Preparation of CuO Nanowires/Ag Composite Substrate and Study on SERS Activity. Plasmonics.

[42] Kurouski D., Postiglione T., Deckert-Gaudig T., Deckert V., Lednev I. (2013). Amide I vibrational mode suppression in surface (SERS) and tip (TERS) enhanced Raman spectra of protein specimens. Analyst.

[43] Chatterley A., Laity P., Holland C., Weidner T., Woutersen S., Giubertoni G. (2022). Broadband Multidimensional Spectroscopy Identifies the Amide II Vibrations in Silkworm Films. Molecules.

[44] Notingher I. (2007). Raman Spectroscopy Cell-based Biosensors. Sensors.

[45] Anastasopoulos J., Beobide A.S., Manikas A., Voyiatzis G. (2017). Quantitative Surface-enhanced Resonance Raman Scattering Analysis of Methylene Blue Using Silver Colloid. J. Raman Spectrosc..

[46] Tycova A., Kleparnik K., Foret F. (2019). Bi-Ligand Modification of Nanoparticles: An Effective Tool for Surface-Enhanced Raman Spectrometry in Salinated Environments. Nanomaterials.

[47] Castro J., López-Ramírez M., Arenas J., Otero J. (2004). Surface-enhanced Raman Scattering of 3-mercaptopropionic Acid Adsorbed on a Colloidal Silver Surface. J. Raman Spectrosc..

[48] Baldrich E., Laczka O., del Campo F., Pascual F.M. (2008). Self-assembled monolayers as a base for immunofunctionalisation: Unequal performance for protein and bacteria detection. Anal. Bioanal. Chem..

[49] Tien-Chun T., Chia-Wei L., Yi-Chen W., Ondevilla A., Osawa M., Hsien-Chang C. (2019). In situ study of EDC/NHS immobilization on gold surface based on attenuated total reflection surface-enhanced infrared absorption spectroscopy (ATR-SEIRAS). Colloids Surf. B Biointerfaces.

[50] Arenas J., Castro J., Otero J., Marcos J. (2001). Study of interaction between aspartic acid and silver by surface-enhanced Raman scattering on H(2)O and D(2)O sols. Biopolymers.

[51] Madzharova F., Heiner Z., Gühlke M., Kneipp J. (2016). Surface-Enhanced Hyper-Raman Spectra of Adenine, Guanine, Cytosine, Thymine, and Uracil. J. Phys. Chem. C Nanomater. Interfaces.

[52] Yoshimoto T., Seki M., Okabe H., Matsuda N., Wu D.-Y., Futamata M. (2022). Three distinct adsorbed states of adenine on gold nanoparticles depending on pH in aqueous solutions. Chem. Phys. Lett..

[53] Costas C., López-Puente V., Bodelón G., González-Bello C., Pérez-Juste J., Pastoriza-Santos I., Liz-Marzán L. (2015). Using Surface Enhanced Raman Scattering to Analyze the Interactions of Protein Receptors with Bacterial Quorum Sensing Modulators. ACS Nano.

[54] Pichardo-Molina J., Frausto-Reyes C., Barbosa-García O., Huerta-Franco R., González-Trujillo J., Ramírez-Alvarado C., Gutiérrez-Juárez G., Medina-Gutiérrez C. (2007). Raman Spectroscopy and Multivariate Analysis of Serum Samples from Breast Cancer Patients. Lasers Med. Sci..

[55] Dingari N., Horowitz G., Kang J., Dasari R. (2012). Raman Spectroscopy Provides a Powerful Diagnostic Tool for Accurate Determination of Albumin Glycation. PLoS ONE.

[56] Wang S., Ye D., Wang B., Xie C. (2020). The Expressions of Keratins and P63 in Primary Squamous Cell Carcinoma of the Thyroid Gland: An Application of Raman Spectroscopy. Onco. Targets. Ther..

[57] Wiggins T., Kumar S., Markar S., Antonowicz S., Hanna G. (2015). Tyrosine, Phenylalanine, and Tryptophan in Gastroesophageal Malignancy: A Systematic Review. Cancer Epidemiol. Biomark. Prev..

[58] Rockberg J., Uhlen M. (2009). Prediction of antibody response using recombinant human protein fragments as antigen. Protein Sci..

[59] Madzharova F., Heiner Z., Kneipp J. (2017). Surface Enhanced Hyper-Raman Scattering of the Amino Acids Tryptophan, Histidine, Phenylalanine, and Tyrosine. J. Phys. Chem. C.

[60] Anfossi L., Baggiani C., Giovannoli C., Giraudi G. (2009). Homogeneous immunoassay based on gold nanoparticles and visible absorption detection. Anal. Bioanal. Chem..

[61] Zhang C., Zhan Y., Wang X., Ming Z., Hong Z. (2003). Hyper-Rayleigh scattering of protein-modified gold nanoparticles. Anal. Biochem..

[62] Pang J., Li P., He H., Xu S., Liu Z. (2022). Molecularly Imprinted Polymers Outperform Lectin Counterparts and Enable More Precise Cancer Diagnosis. Chem. Sci..

[63] Pettinato G., Coughlan M., Zhang X., Chen L., Khan U., Glyavina M., Sheil C., Upputuri P., Zakharov Y., Vitkin E. (2021). Spectroscopic Label-Free Microscopy of Changes in Live Cell Chromatin and Biochemical Composition in Transplantable Organoids. Sci. Adv.

[64] Zhang Y., Cheng M., Wang Y., Zhang J., Hua Z. (2021). A SERS Biosensor Regulated by Tilt Angle: An Immunochip for α-Fetoprotein. J. Mater. Sci..

[65] Liu J., Geng Q., Geng Z. (2024). A Route to the Colorimetric Detection of Alpha-Fetoprotein Based on a Smartphone. Micromachines.

[66] Li L., Liu C., Cao X., Tan L., Lu W. (2017). Multiplexing Determination of Cancer-Associated Biomarkers by Surface-Enhanced Raman Scattering Using Ordered Gold Nanohoneycomb Arrays. Bioanalysis.

[67] Wu Y., Yi R., Zang H., Li J., Xu R., Zhao F., Wang J., Fu C., Chen J. (2023). A ratiometric SERS sensor with one signal probe for ultrasensitive and quantitative monitoring of serum xanthine. Analyst.

[68] Wang S., Qin Y., Zou Z. (2016). Determination of Liver Cancer Biomarkers by Surface-Enhanced Raman Scattering Using Gold-Silica Nanoparticles. Anal. Lett..

[69] Zhang Y., Sun H., Gao R., Zhang F., Zhu A., Chen L., Wang Y. (2018). Facile SERS-active chip (PS@Ag/SiO_2_/Ag) for the determination of HCC biomarker. Sens. Actuators B Chem..

[70] Wu M., Hartanto H., Wu S., Jiang T., Wang G., Chen T.-H. (2023). Visualizing alpha-fetoprotein level in undiluted serum based on microfluidic particle accumulation. Sens. Actuators B Chem..

[71] Becerril-Castro I., Calderon I., Pazos-Perez N., Guerrini L., Schulz F., Feliu N., Chakraborty I., Giannini V., Parak W., Alvarez-Puebla R. (2022). Gold Nanostars: Synthesis, Optical and SERS Analytical Properties. Anal. Sens..

[72] García-Ramírez J., Castillo M., Santiago E., Torres J., Rodríguez A., Aguilar E., Hernández I., Dupont P., Zamora-Peredo L. (2025). Detection of MB and BSA with Au–Ag Nanostar-Coated Microspheres. MRS Adv..

